# Practical guide for microscopic identification of infectious gastrointestinal nematode larvae in sheep from Sardinia, Italy, backed by molecular analysis

**DOI:** 10.1186/s13071-021-05013-9

**Published:** 2021-09-28

**Authors:** Stephane Knoll, Giorgia Dessì, Claudia Tamponi, Luisa Meloni, Lia Cavallo, Naunain Mehmood, Philippe Jacquiet, Antonio Scala, Maria Grazia Cappai, Antonio Varcasia

**Affiliations:** 1 Dipartimento di Medicina Veterinaria, Università di Sassari, Sassari, Italy; 2 Department of Zoology, University of Sargodha, Sargodha, Pakistan; 3 Laboratoire de Parasitologie, École Nationale Vétérinaire de Toulouse, Toulouse, France

**Keywords:** Gastrointestinal nematodes, Larvae, Larval culture, Morphology, Morphometry, Sheep

## Abstract

**Background:**

Gastrointestinal nematodes (GIN) are ubiquitous in small ruminant farming, representing a major health and production concern. Given their differences in pathogenicity and the current problems regarding anthelmintic resistance, specific diagnosis of GIN is of significant importance. At present, the most widely applied method for this entails culture and microscopic analysis of third-stage larvae, allowing for identification at least to the genus level. Overall, a variety of keys for microscopic analysis have been published, showing substantial variation. Given this fact, this study aimed to produce a practical and updated guide for the identification of infective ovine GIN larvae.

**Methods:**

Using existing keys and protocols, a total of 173larvae of the most common species/genera of ovine GIN from pooled faecal samples from Sardinia (Italy) were identified and extracted, and further individual molecular identification was performed. Morphometric and morphological data as well as high-quality photographs were collected and combined to produce the final guide.

**Results:**

GIN microscopically and molecularly identified during this research include *Trichostrongylus* spp., *Teladorsagia circumcincta*, *Haemonchus contortus*, *Cooperia curticei*, and *Chabertia ovina.* Based on microscopic analysis, 73.5% of the larvae were correctly identified. Based on sheathed tail length, 91.8% were correctly classified into their respective preliminary groups.

**Conclusions:**

It is crucial for the microscopic identification of infectious GIN larvae to examine each larva in its entirety and thus to take multiple characteristics into account to obtain an accurate diagnosis. However, a preliminary classification based on sheathed tail length (resulting in three groups: A, short; B, medium; C, long) was found to be effective. Further identification within group A can be achieved based on the presence of a cranial inflexion, caudal tubercles and full body measurements (*Trichostrongylus* spp. < 720 µm, *T. circumcincta* ≥ 720 µm). Larvae within group B can be differentiated based on sheathed tail morphometry (*H. contortus* > 65 µm, *C. curticei* ≤ 65 µm), the presence of cranial refractile bodies, total body length measurements (*H. contortus* ≤ 790 µm, *C. curticei* > 790 µm) and shape of the cranial extremity. Finally, all characteristics proposed for the differentiation between *Oesophagostomum* spp. and *C. ovina* larvae (group C) were found to have considerable restrictions.

**Graphical abstract:**

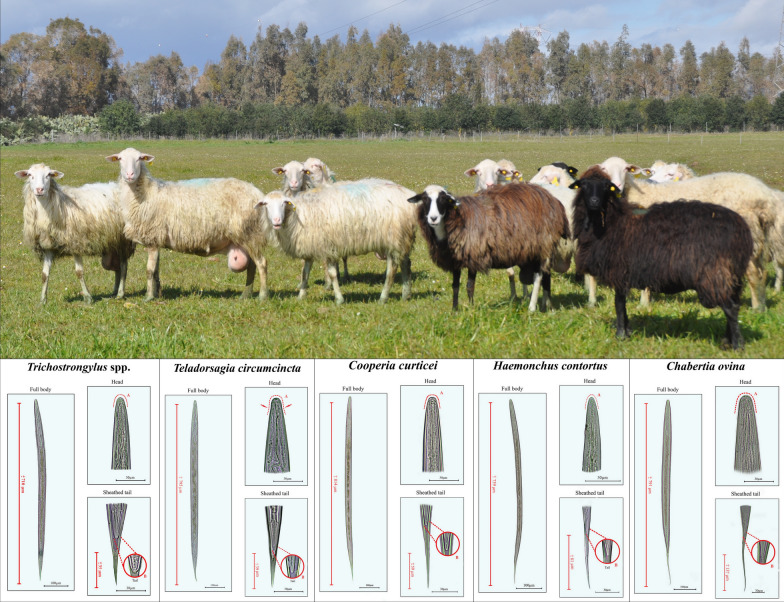

**Supplementary Information:**

The online version contains supplementary material available at 10.1186/s13071-021-05013-9.

## Background

Gastrointestinal nematodes (GIN) represent one of the major health and production concerns in sheep farming worldwide. In general, clinical and subclinical infections with these worms can, through their direct and indirect effects on the gastrointestinal tract of animals, cause morbidity and mortality, leading to significant milk, wool and meat production losses [1–5]. In fact, in several key sheep farming countries, GIN infections have been identified as the most costly common disease in the industry [6, 7].

In domestic sheep, frequently encountered GIN belong to the strongyle group and include *Trichostrongylus* spp., *Teladorsagia circumcincta*, *Haemonchus contortus*, *Cooperia* spp., *Oesophagostomum* spp., *Chabertia ovina*, *Bunostomum trigonocephalum*, and *Nematodirus* spp., and almost all individuals are infected with one or more of these species [2, 8, 9].

Depending on the worm burden and species, clinical signs of parasitosis with GIN in sheep include anorexia, diarrhoea, anemia, oedema, reduced fertility and increased susceptibility to other pathogens [2, 3, 9–11]. However, many of the common nematode genera differ substantially in pathogenicity, making specific diagnosis essential in case of morbidity [8, 12]. Additionally, overuse and improper use of anthelmintic drugs over the years has led to increasing problems of anthelmintic resistance (especially in small ruminant farming) [2, 13–15], further reinforcing the need for specific diagnosis of the infecting nematodes. Consequently, correct identification of GIN has become increasingly important in the development of effective control and prevention strategies in sustainable sheep farming worldwide [7, 8, 16, 17]. Specific identification is also crucial within the context of parasite biological, ecological and epidemiological research in general [17, 18].

Routine diagnostic techniques developed to date do not allow for the specific diagnosis of GIN present [2, 17]. Overall, standard diagnostics for the evaluation of GIN infections in livestock are performed using relatively inaccurate methods, such as microscopic examination and faecal egg counts (either through the McMaster or flotation technique), which do not allow for differentiation between eggs of similar shape and size [2, 17, 19, 20], with only a few exceptions, such as *Strongyloides papillosus*, *Nematodirus* spp., *Trichuris* spp., *Capillaria* spp., and *Marshallagia* spp. [12, 20].

Although significant advancements in molecular diagnostics for the identification of livestock nematodes have been made within the last decade [2, 7, 8, 16–18, 21], microscopic identification of larvae still represents the most widely applied method for specific nematode diagnosis in parasitology laboratories [8, 11]. In this regard, through the recognition of a handful of distinguishing morphological characteristics, with or without the performance of microscopic measurement, infectious third-stage larvae (L_3_) from cultured faecal samples are identifiable at least to the genus level [7, 12, 19, 20, 22].

However, microscopic identification poses some challenges, including time- and labor-intensive protocols and reliance on specialist experience and expertise, as many of the differences between GIN genera are very small [7, 12, 16, 17]. Additionally, within the current scientific literature, significant overlap between length characteristics of different GIN genera and substantial variation within the species are reported. Besides, morphometric characteristics (e.g., length parameters) of nematode larvae have been shown to differ based on environmental factors, including climate [7, 22, 23].

To date, various protocols and keys for the identification of GIN L_3_ in sheep have been produced, including for the northern United States, Australia, the United Kingdom, the French-Atlantic regions, New Zealand and South Africa [12, 19, 22, 24–27]. So far, no specific guidelines for the identification of Mediterranean and South European GIN larvae have been published. Therefore, and given the variability of the published microscopic data, a survey combining classical parasitological identification (based on morphometric and morphological features) with molecular analysis (polymerase chain reaction [PCR] and DNA sequencing) was carried out on faecal samples from Sardinia, Italy, where over three million sheep are bred following extensive management. The aim of this study is to produce a practical guide as well as to provide updated keys for the identification of the most common infectious GIN larvae in sheep.

## Methods

### Study area and sampling

The island of Sardinia, representing one of the 20 regions of Italy, is located directly south of the French island of Corsica and west of mainland Italy. As the second largest island in the Mediterranean Sea, Sardinia is characterized by hot and dry summers with mild and wet winters [28]. The island holds the highest number of sheep farms in Italy, with more than 45% of the Italian sheep population (over three million head of sheep). Hence, Sardinia alone is responsible for 25% of total EU-27 sheep milk production. Furthermore, within Sardinia, Sassari province has the second highest sheep count on the island (22.8% of the total Sardinian population) [29, 30].

Faecal samples included in this research were obtained during routine diagnostic activities of the Parasitology Laboratory of the Department of Veterinary Medicine of the University of Sassari and included samples from both replacements and adult Sarda sheep. In order to avoid interference of the growth of GIN larvae, farms were considered for which no anthelmintic treatment was carried out in the 3 months preceding the copromicroscopic analysis. In detail, rectal faecal samples were collected and stored in refrigerated containers for transport. Upon arrival, faecal pools consisting of five randomly selected animals per farm were subjected to coprological examination through the FLOTAC^®^ method using a zinc sulphate solution (specific gravity 1.35), and eggs per gram (EPG) were obtained (level of sensitivity: 15 EPG) [31, 32]. Fifteen pools with GIN EPG values higher than 150 were selected to set up faecal cultures, ensuring the presence of sufficient GIN larvae for microscopic identification. For each farm, faecal collection, coprological examination and the set-up of faecal cultures occurred within the same workday.

### Faecal culture and harvesting of infective larvae

Faecal culture was performed immediately following coprological examination according to previously described protocols [33, 34]. Briefly, 50 g of each selected pooled sample was placed in 500 ml plastic cylindrical jars without a cap (Kartell S.p.A., Milan, Italy) and incubated at 22–25 °C for 10 days in a dark cabinet and moistened daily by spraying with water.

After incubation, the plastic container was filled with lukewarm tap water and overturned in a petri dish. Forty millilitres of lukewarm tap water was added to the petri dish and the whole exposed to light for 24 h, stimulating the hatched larvae to migrate out of the plastic container into the petri dish. The water containing L_3_ was collected in a 50 ml falcon tube and stored at 4 °C for up to 6 months.

### Single larva selection and microscopic identification

A total of 30 individual L_3_ for each morphologically distinguishable species/genus of ovine GIN encountered were identified and extracted. Possible species/genera encountered were *Trichostrongylus* spp., *T. circumcincta*, *H. contortus*, *Cooperia* spp., *Oesophagostomum* spp. and *C. ovina*. Other nematode species such as *Nematodirus* spp. and *Trichuris* spp., found through the FLOTAC^®^ method, were not included in this research, as they required different larval cultivation protocols from the one applied here.

For larval selection, 10 µl of larval solution was pipetted onto a glass microscope slide, creating individual droplets (~ 2 µl). Droplets were inspected for the presence of larvae at ×50 magnification, and those containing a single larva were covered by a 4 × 4 mm microscope coverslip. The whole microscope slide was briefly subjected to an open flame, effectively killing the larvae and ensuring an erect position for optimal identification. Following this procedure, photographs of each larva’s full body, caudal extremity (tail) and cranial extremity (head) were taken. In addition, measurements of the larva’s full body (tip of cranial extremity to tip of sheathed tail), sheathed tail (tail base to tip of sheathed tail) and tail filament (sheathed tail filament transition to sheathed tail tip) were recorded. All photographs and measurements were taken at ×400 magnification except for the larva’s full body, which was taken at ×100 magnification. Additionally, notes were taken regarding the shape of the larva’s tail, sheathed tail and head, as well as its intestinal cells when possible. Photographs and measurements were taken using a Digital C-mount Camera (S/N 2026100119 TP8000UHD 4 K Color CMOS) and matching software (alexasoft.com).

Larvae were identified using a combination of the morphometric and morphological keys published by I.N.R.A. [27], van Wyk [19], Zajac and Conboy [20], and van Wyk and Mayhew [13]. A summary of the morphometric and morphological characteristics utilized can be found in Table 1.

**Table 1:** Morphometric and morphological features reported for the microscopic identification of the third-stage larvae of the gastrointestinal nematodes (GIN) of sheep

Species/genus	Full body length (µm)	Sheathed tail length (µm)	Group (sheathed tail)	Number of intestinal cells	Shape and configuration of intestinal cells		Shape of head	Shape of tail base	Shape of sheathed tail	Presence of sheathed tail filament (proportion of sheathed tail)
*Trichostrongylus* spp.	693–714^a^	25–29a	Short (A)	16	Triangular, 1 terminal cell		Rounded	1–3 tubercles or smooth	Conic	No
	622–796^b^	21–40^b^								
	710^c^	18–31^c^								
*Teladorsagia circumcincta*	819–907^a^	32–46^a^	Short (A)	16	Triangular/pentagonal, 1 terminal cell		Flat/square, cranial inflexion	Smooth	Blunt	No
	797–910^b^	30–60^b^								
	830^c^	30–44^c^								
*Haemonchus contortus*	657–733^a^	40–80^a^	Medium (B)	16	2 terminal cells		Bullet–shaped	Smooth and pointed	Slightly curved base	Yes (small, 10–15%^c^)
	650–751^b^	65–78^b^								
	730^c^	65–82^c^								
*Cooperia* spp.	752–862^a^	74^a^	Medium (B)	16	Triangular, 1 terminal cell		Square/rounded, 2 refractile bodies	Smooth	Pointed	Yes (20%^c^)
	711–924^b^	35–82^b^								
	865^c^	39–82^c^								
*Oesophagostomum* spp.	796–940^a^	130–170^a^	Long (C)	24–32^a^	Triangular/pentagonal		Rounded^b^	Smooth	/	Yes (long, 60–70%^c^)
	771–923^b^	125–160^b^		16–24^b^			Square^c^			
	850^c^	122–207^c^		18–22^c^						
*Chabertia ovina*	747–800^a^	105–120^a^	Long (C)	24–32^a^	Rectangular		Rounded^b^	Smooth	/	Yes (long, 25%^c^)
	710–789^b^	110–150^b^		24–32^b^			Square^c^			
	750^c^	101–150^*c^		28–32^c^						

Data acquired from the following sources: I.N.R.A. [27]^a^, Zajac and Conboy [20]^b^ and van Wyk [19] and van Wyk and Mayhew [13]^c^ . No specific reference was placed in the table if all sources agree

**Fig. 1:**
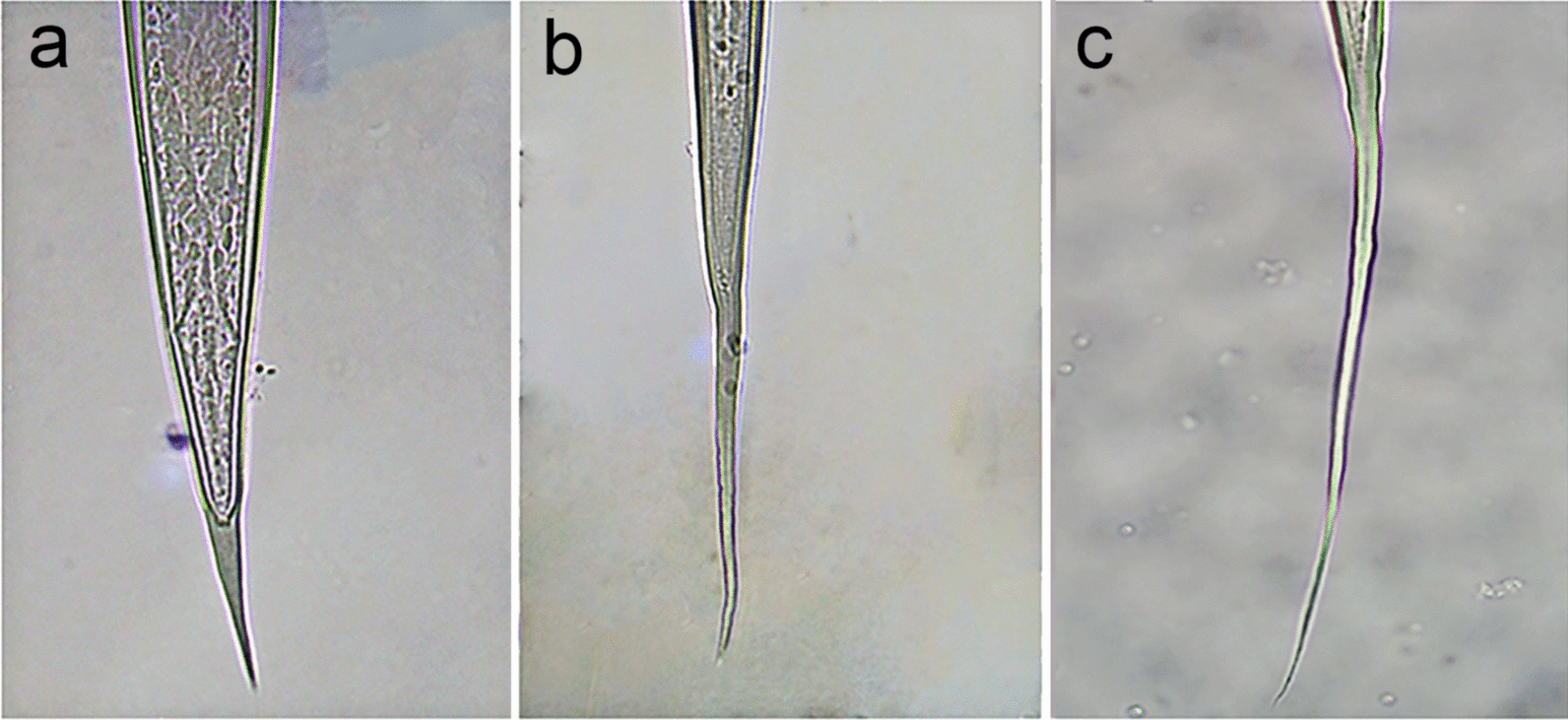
Preliminary classification method for the infectious larvae of the common gastrointestinal nematodes of sheep based on sheathed tail length. Three groups: **a** short sheathed tail (25–50 µm), **b** medium sheathed tail (51–89 µm) and **c** long sheathed tail (90–200 µm)

As a primary means of classification, selected larvae were categorized into three groups based on their sheathed tail length (Fig. 1) [35]: A, short sheathed tail (25–50 µm) including *Trichostrongylus* spp. and *T. circumcincta*; B, medium sheathed tail (51–89 µm) including *Cooperia* spp. and *H. contortus*; and C, long sheathed tail (90–200 µm) including *C. ovina* and *Oesophagostomum* spp. Morphological characteristics used for further identification within each group were as follows: 1, full body length; 2, number of intestinal cells; 3, shape and configuration of intestinal cells; 4, shape of head; 5, shape of tail base; 6, shape of sheathed tail; 7, presence and length of sheathed tail filament.

Following microscopic identification, microscope coverslips were removed and individual larvae collected. Each larva was carefully picked up using a fine sterile syringe needle (28 G) and flushed into a sterile 1.5 ml Eppendorf tube using 200 µl of PBS solution and stored at 4 °C until molecular examination.

All actions described above were carried out by a single researcher.

### DNA extraction, amplification and sequencing

DNA from the single GIN L_3_ isolated was extracted using the G-spin™ Total DNA Extraction Kit (iNtRON Biotechnology^©^, South Korea) following the manufacturer’s instructions. Following extraction, DNA concentration was assessed by a NanoDrop^©^ Lite spectrophotometer and subsequently stored at −20 °C until DNA amplification. PCR was performed using the primers NC1 and NC2 to amplify a 311–331-bp fragment encompassing the ITS-2 and partial flanking regions of the 5.8S and 28S rRNA genes [21]. PCR reaction was carried out in 25 µl total volume containing 10 × PCR buffer, 1.5 mM MgCl_2_, 0.2 mM of every deoxynucleotide triphosphate (dNTP), 0.2 µM of each primer, 1.25 U of Taq (Thermo Fisher Scientific, MA, USA) and 5 µl of genomic DNA. The thermal cycler conditions were 95 °C for 5 min, followed by 35 cycles of 95 °C for 30 s, 54 °C for 30 s, 68 °C for 1 min and a final extension of 68 °C for 1 min [36].

Following PCR, products were resolved on 2% agarose gel electrophoresis (Cleaver Scientific^©^ Agarose CSL-AG500) and visualized by UVIdoc HD2 (UVITEC, Cambridge, UK). Positive samples were purified using NucleoSpin Gel and PCR Clean-Up (Macherey–Nagel GmbH & Co. KG, Düren, Germany) and sent to an external sequencing service (Eurofins Genomics, Ebersberg, Germany).

### Data collection and analysis

Data were collected on a spreadsheet (Microsoft Excel^®^), and measurements processed using R™ (R i386 3.6.2) and RStudio™ software.

The ITS-2 and partial flanking regions of the 5.8S and 28S rRNA nucleotide sequences were run through the Basic Local Alignment Search Tool (BLAST™, https://blast.ncbi.nlm.nih.gov/Blast.cgi) to look up the reference sequences for each GIN species. A sequence identity threshold of > 99% was chosen for the assignment of each obtained nucleotide sequence to its respective reference sequence. The sequence chromatograms were checked individually through the FinchTV viewer (Geospiza, Inc., Seattle, WA, USA) and were subjected to multiple alignment along the reference sequences. The sequences obtained for each species in this study were deposited in the GenBank database (Additional file 1: Table S1).

Results of the molecular identification were compared to those obtained through microscopic identification to ascertain the accuracy of the existing morphometric and morphological keys. In the case of correct identification, acquired measurements and characteristics were accepted as representative for said species/genus within a Mediterranean and southern European climate. In the case of incorrect identification, morphological characteristics (through photographs) and measurements were revised and allocated to the correct species/genus.

For comparison of average sheathed tail and full body length between groups (A, B, C) and between species/genera (*Trichostrongylus* spp., *T. circumcincta*, *Cooperia* spp., *H. contortus*, *C. ovina*) identified by molecular methods, analysis of variance (ANOVA) was performed and was followed by the Tukey test for post hoc analysis. A difference of 30 µm and 10 µm in total body length and sheathed tail length, respectively, were considered as relevant within the context of microscopic identification. Acquired results were considered statistically significant at a *P*-value less than 0.05 (*P* < 0.05).

## Results

Faecal culture revealed the presence of a mixed GIN population for each of the 15 samples, consisting of larvae belonging to at least two sheathed tail groups.

Based on morphometric characteristics, a total of 173 individual larvae were extracted and classified as *Trichostrongylus* spp. (*n* = 30), *T. circumcincta* (*n* = 30), *Cooperia* spp. (*n* = 30), *H. contortus* (*n* = 30), *C. ovina* (*n* = 30) and *Oesophagostomum* spp. (*n* = 23).

DNA amplification was positive for 67.6% (117/173) of the samples (*Trichostrongylus* spp., 22/30; *T. circumcincta*, 22/30; *Cooperia* spp., 23/30; *H. contortus*, 22/30; *C. ovina*, 16/30; *Oesophagostomum* spp., 12/23). DNA sequencing was successful for 83.8% (98/117) of the samples and revealed the presence of 24 *Trichostrongylus* spp., 10 T*. circumcincta*, 22 *Cooperia* spp., 24 *H. contortus*, 18 *C. ovina* and zero *Oesophagostomum* spp.

Based on the BLAST algorithm, three species were identified within the genus *Trichostrongylus*, i.e. *Trichostrongylus colubriformis* (14/24, 58.3%), *Trichostrongylus vitrinus* (5/24, 20.8%) and *Trichostrongylus axei* (5/24, 20.8%), having an identity matching 100% to the ITS-2 and partial flanking regions of the 5.8S and 28S rRNA sequences in the GenBank database with the accession numbers KY355062 and KC521364 (*T. colubriformis*), KC998732 and MK271675 (*T. vitrinus*), and AY439026 and MK936874 (*T. axei*). The nucleotide sequences for *T. circumcincta* were 100% identical to its reference sequences (JQ989274 and KF989498). ITS-2 and partial flanking regions of the 5.8S and 28S rRNA sequences for the genus *Cooperia* revealed the presence of a single species, *Cooperia curticei* (22/22, 100%), having a matching sequence identity of 99.3% to the nucleotide sequences (KC998738 and KC998736) deposited in the NCBI database. The sequences for *H. contortus* were 100% identical to the nucleotide sequences with the accession numbers MN845169 and MF398432. For *C. ovina*, BLAST search yielded 100% homology to the two sequences (accession numbers: KJ420887 and KF913470). A complete sample list including microscopic and molecular identification as well as the respective accession numbers for each species (MZ323365-MZ323371) can be found in Additional file 1: Table S1.

Based on sheathed tail length, 91.8% (90/98) of larvae were correctly assigned to their respective preliminary groups. Within group A, 94.4% (34/36) of the samples were correctly classified, with two larvae belonging to group B. In group B, 100% (38/38) of the larvae were correctly classified. For group C, 75.0% (18/24) of the samples were correctly classified, with six larvae belonging to group B. In the end, based on genetic analysis, 34 larvae were found to belong to group A, 46 to group B and 18 to group C.

Based on microscopic analysis, 73.5% (72/98) of the larvae were correctly identified to the species/genus level. For *Trichostrongylus* spp. and *H. contortus*, 100% of the larvae were correctly identified (*Trichostrongylus* spp., 17/17; *H. contortus*, 16/16). For *T. circumcincta*, 52.6% (10/19) of the samples were correctly identified, with seven larvae belonging to *Trichostrongylus* spp. and two to *Cooperia* spp. For *Cooperia* spp. and *C. ovina*, 90.9% (20/22) and 60.0% (9/15) of the larvae were correctly identified, respectively, with all larvae that were wrongly identified in fact belonging to *H. contortus.* Finally, 100% (9/9) of the *Oesophagostomum* spp. samples were wrongly identified and turned out to be *C. ovina* larvae.

A summary of the relationship between microscopic identification and molecular analysis of the samples is given in Table 2, and an overview of the characteristic morphometric and morphological features (microscopic keys) of each GIN species/genus encountered within this research is given in Table 3. Additionally, summary images of infective larvae of the GIN species/genera encountered within this research can be found in Additional file 2: Figures S1–S5.

**Table 2:** Summary of the relationship between microscopic identification and molecular analysis of the individual gastrointestinal nematode (GIN) larvae examined in the present study

Microscopic identification	Number of samples	Successfully amplified (PCR)	Successfully sequenced	Correctly identified	Molecular identification	Number of samples
*Trichostrongylus* spp.	30	22	17	100%	*Trichostrongylus colubriformis*	12
					*Trichostrongylus vitrinus*	3
					*Trichostrongylus axei*	2
*Teladorsagia circumcincta*	30	22	19	52.6%	*T. circumcincta*	10
					*T. colubriformis*	2
					*T. vitrinus*	2
					*T. axei*	3
					*Cooperia curticei*	2
*Haemonchus contortus*	30	22	16	100%	*H. contortus*	16
*Cooperia* spp.	30	23	22	90.9%	*C. curticei*	20
					*H. contortus*	2
*Chabertia ovina*	30	16	15	60.0%	*C. ovina*	9
					*H. contortus*	6
*Oesophagostomum* spp.	23	12	9	0%	*C. ovina*	9

**Table 3:** Morphometric and morphological features of the third-stage larvae of the GIN of sheep observed in the present study using DNA analysis as the gold standard

Species/genus	Mean full body length (range, µm)	Standard deviation (µm)	Mean sheathed tail length (range, µm)	Standard deviation (µm)	Group (sheathed tail)	Shape of head	Shape of tail base	Shape of sheathed tail	Presence of sheathed tail filament
*Trichostrongylus* spp.	709	61	35	5	Short (A)	Rounded	1–3 tubercles or smooth	Conic	No
	(620–863)		(27–55)						
*Teladorsagia circumcincta*	795	52	39	3	Short (A)	Flat/square, cranial inflexion	Smooth	Blunt	No
	(740–902)		(36–45)						
*Haemonchus contortus*	739	29	81	6	Medium (B)	Bullet-shaped	Smooth and pointed	Slightly curved base	?^a^
	(669–817)		(67–91)						
*Cooperia curticei*	814	39	59	5	Medium (B)	Square/rounded, 2 refractile bodies	Smooth	Pointed	?^a^
	(739–886)		(50–68)						
*Chabertia ovina*	791	37	137	17	Long (C)	Rounded	Smooth	–	Yes, long
	(722–848)		(86–160)						

^a^The presence of a sheathed tail filament for larvae classified within group B was unclear in our hands

Statistical analysis revealed the three preliminary classification groups (A, B and C) to have significantly different sheathed tail lengths (*F*_(2, 95)_ = 440.6, *P* < 0.001). Post hoc analysis showed significant differences between all three groups (A–B: *P* < 0.001, A–C: *P* < 0.001, B–C: *P* < 0.001). ANOVA on the sheathed tail length of all species/genera showed significant differences between these larvae (*F*_(4, 93)_ = 425.7, *P* < 0.001). Post hoc analysis showed the sheathed tail length of *H. contortus*, *Cooperia* spp. and *C. ovina* to be significantly different from each other and from *Trichostrongylus* spp. and *T. circumcincta* (*P* < 0.001). The sheathed tail length of *Trichostrongylus* spp. and *T. circumcincta* were not found to be significantly different from each other (*P* = 0.748).

A significant difference was found between the total body length of the larvae of the different preliminary classification groups (*F*_(2, 95)_ = 7.454, *P* = 0.001). Post hoc analysis showed significant differences between group A and both other groups (A–B: *P* = 0.003, A–C: *P* = 0.006) and no difference between groups B and C (*P* = 0.564). A significant difference in body length between the different species/genera within this research was also encountered (*F*_(4, 93)_ = 20.63, *P* < 0.001). Post hoc analysis showed *Trichostrongylus* spp. larvae to be significantly different in full body length from *T. circumcincta* (*P* < 0.001), *Cooperia* spp. (*P* < 0.001) and *C. ovina* (*P* < 0.001) larvae. No significant difference was found compared to *H. contortus* larvae (*P* = 0.156). *Haemonchus contortus* larvae were different in body length from *C. ovina* (*P* = 0.003), *Cooperia* spp. (*P* < 0.001) and *T. circumcincta* (*P* = 0.009) larvae. Larvae belonging to *Cooperia* spp. did not differ significantly in full body length compared to *T. circumcincta* (*P* = 0.820), and finally, *T. circumcincta* and *Cooperia* spp. were found not to have significantly different full body lengths compared to *C. ovina* larvae (*P* = 0.486, *P* = 0.999).

**Fig. 2:**
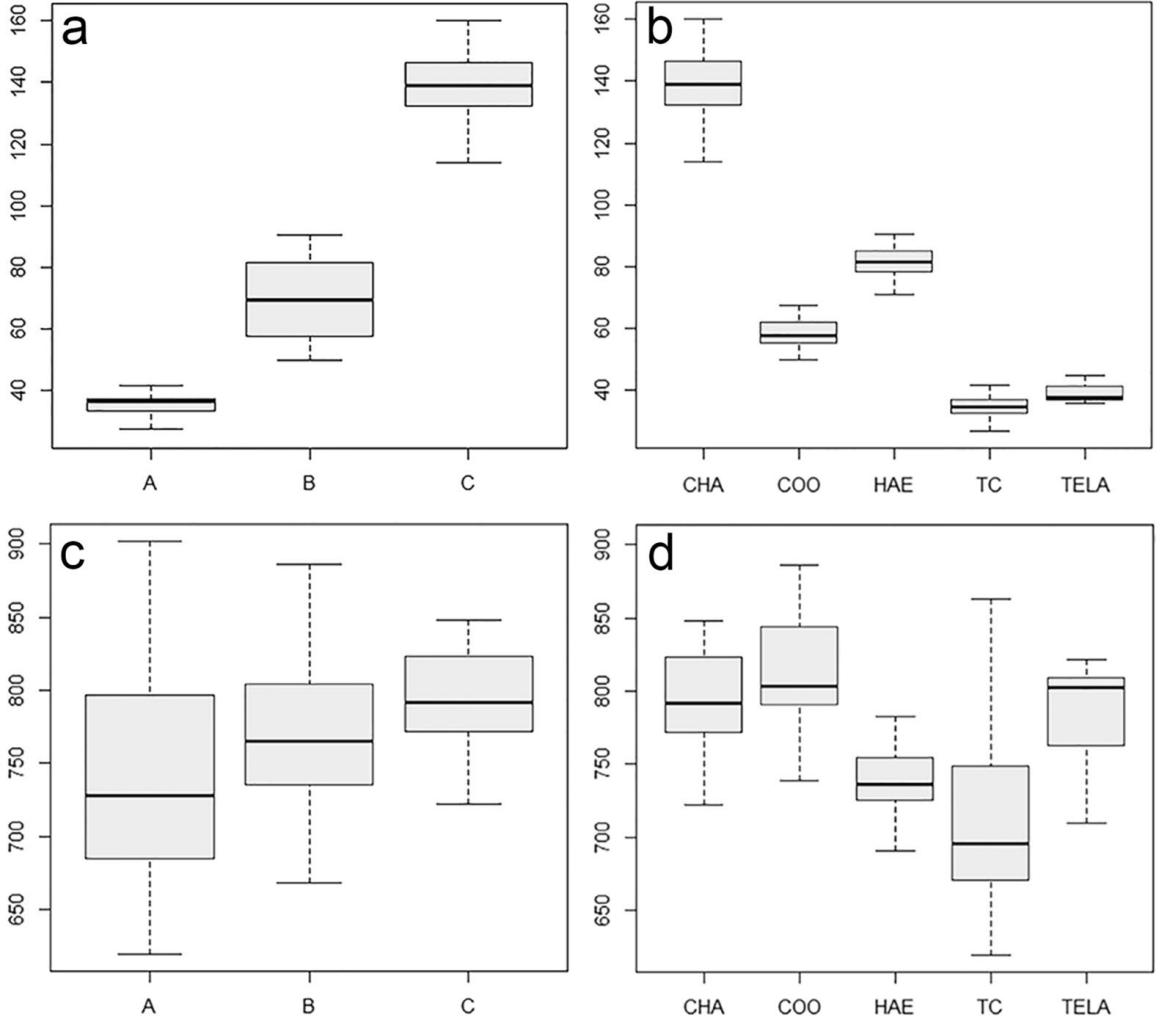
Boxplots of the mean sheathed tail length per group (**a**) and per species/genus (**b**) and the full body length per group (**c**) and per species/genus (**d**) of the infectious larvae of gastrointestinal nematodes of sheep identified through molecular methods within this research. Dark lines represent the mean value and bars represent the standard deviation for each respective group/species/genus. Group A: short sheathed tail (*Trichostrongylus* spp. and *Teladorsagia circumcincta*), group B: medium sheathed tail (*Haemonchus contortus* and *Cooperia curticei*), group C: long sheathed tail (*Chabertia ovina*). Abbreviations: TC, *Trichostrongylus* spp.; TELA, *Teladorsagia circumcincta*; HAE, *Haemonchus contortus*; COO, *Cooperia curticei*; CHA, *Chabertia ovina*

Summary boxplots of the mean sheathed tail length and the full body length per group and per species/genus identified through molecular methods are given in Fig. 2.

## Discussion

This research reports, for the first time, validation of microscopic identification keys of single infectious ovine GIN larvae from faecal samples (containing multiple species) through molecular analysis. The authors verified the identity of each extracted larvae, offering a unique perspective into the accuracy of existing morphometric and morphological identification keys. Besides, the novelty of this research can be found in the enhancement of such keys from a practical point of view, providing the most reliable traits for correct L_3_ identification.

Overall, microscopic identification of ovine GIN infectious larvae is best achieved through a combination of both morphology and morphometry. As the size characteristics of different species/genera of GIN larvae overlap, measurements alone are insufficient for accurate identification. Shape of head, tail and sheathed tail are key additional characteristics for the identification of GIN L_3_ [20]. Our findings, as well as current scientific literature, stress the importance of examining L_3_ in their totality for accurate identification and thus not solely relying on one characteristic in particular [7, 12].

**Fig. 3:**
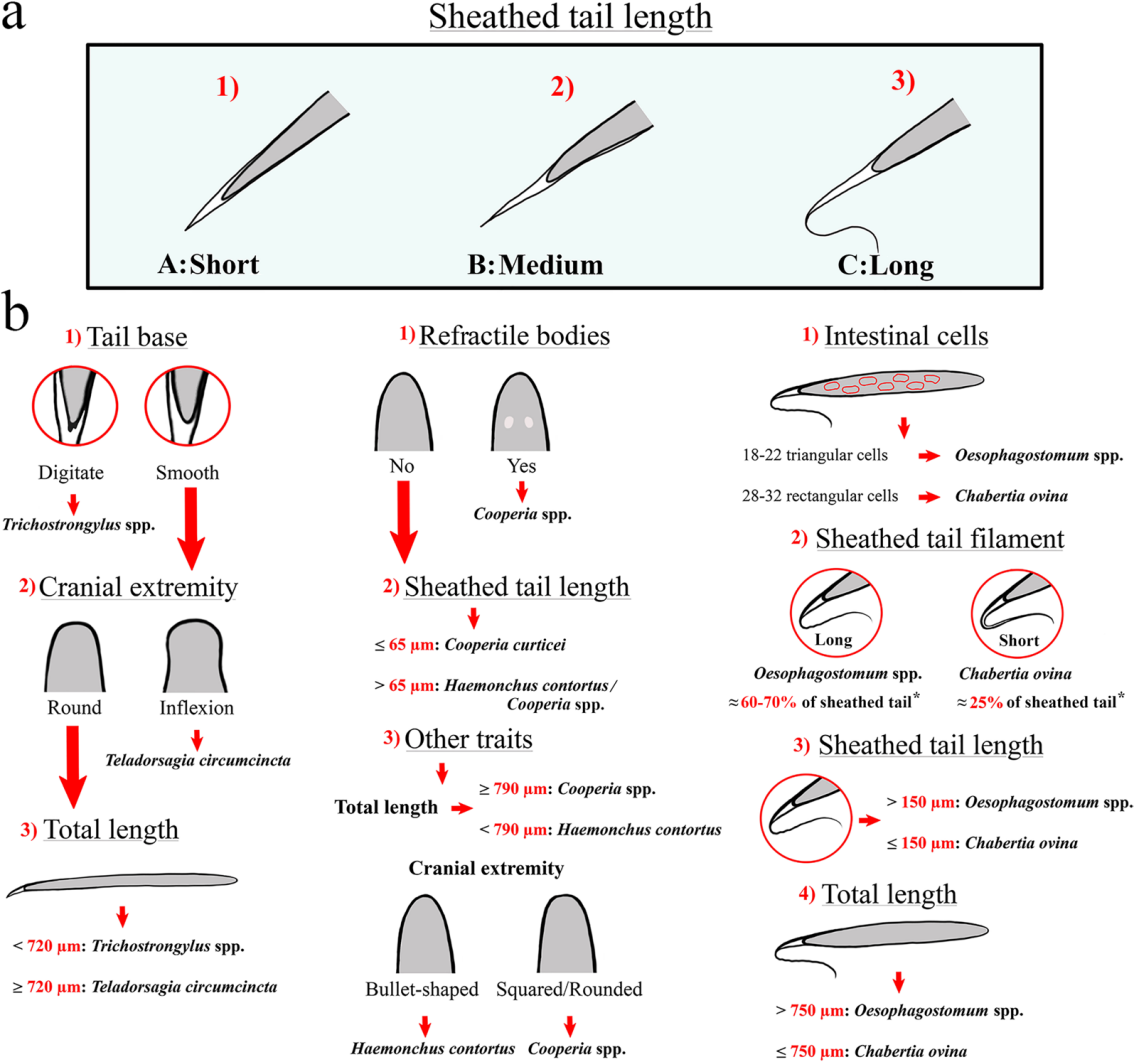
Decision tree for the microscopic identification of the infectious larvae of the common ovine gastrointestinal nematodes (GIN). **a** Preliminary classification based on sheathed tail length, **b** secondary classification (within each respective sheathed tail group: A, B, C) based on additional morphological and morphometric characteristics. *Approximative proportion of the sheathed tail that is filamentous [13]

The authors have found, in accordance with Bowman [35], that the easiest way to identify individual infectious GIN larvae of sheep is firstly based on their sheathed tail length, dividing encountered larvae into three main groups: short (A), *Trichostrongylus* spp. and *T. circumcincta*; medium (B), *Cooperia* spp. and *H. contortus*; and long (C), *C. ovina* and *Oesophagostomum* spp. (Fig. 1). Although morphometric parameters for each group are defined here, with minimal practice, larvae can easily be classified within their respective groups without the performance of microscopic measurement. Further identification of the GIN species/genera within each group is based on microscopic measurements as well as additional morphological characteristics. A global decision tree (based on our findings as well as current scientific literature) for the identification of the infectious larvae of the six common species/genera of ovine GIN is given in Fig. 3.

Gastrointestinal nematode species included within the first group (A, short sheathed tail) are challenging to differentiate as morphological differences between *Trichostrongylus* spp. (Additional file 2: Figure S1) and *T. circumcincta* (Additional file 2: Figure S2) are quite small [12, 17, 19, 22, 25]. In particular, infectious *T. axei* and *T. circumcincta* larvae are very similar, as *T. axei* larvae do not have tubercles on their caudal extremity [22]. Given this fact, misidentification is common and, ultimately, three larvae morphologically classified as being *T. circumcincta* within this research were genetically identified as *T. axei*.

Despite the frequent overlap in total body length between the larvae of the common GIN species in sheep, a rudimentary distinction between *Trichostrongylus* spp. and *T. circumcincta* can be made based on overall size (Fig. 2c). As previously reported [12, 19, 20, 22, 24, 25], and confirmed in this research, full body length of *T. circumcincta* is commonly greater compared to those of *Trichostrongylus* spp. (*Trichostrongylus* spp., 709.43 ± 30.43 µm; *T. circumcincta*, 795.32 ± 25.84 µm). As a general rule, a cut-off of 720 µm can be used for the identification of *T. circumcincta* [25]. Nevertheless, as different species of GIN require (slightly) different developmental conditions (e.g., temperature and moisture) [7, 17, 23], any specific faecal culture protocol could favour the development of one species over another. Although unlikely (based on the continuous reports of size difference between the two genera in question), any size difference here might thus have been the result of more favourable culture conditions for *T. circumcincta*.

**Fig. 4:**
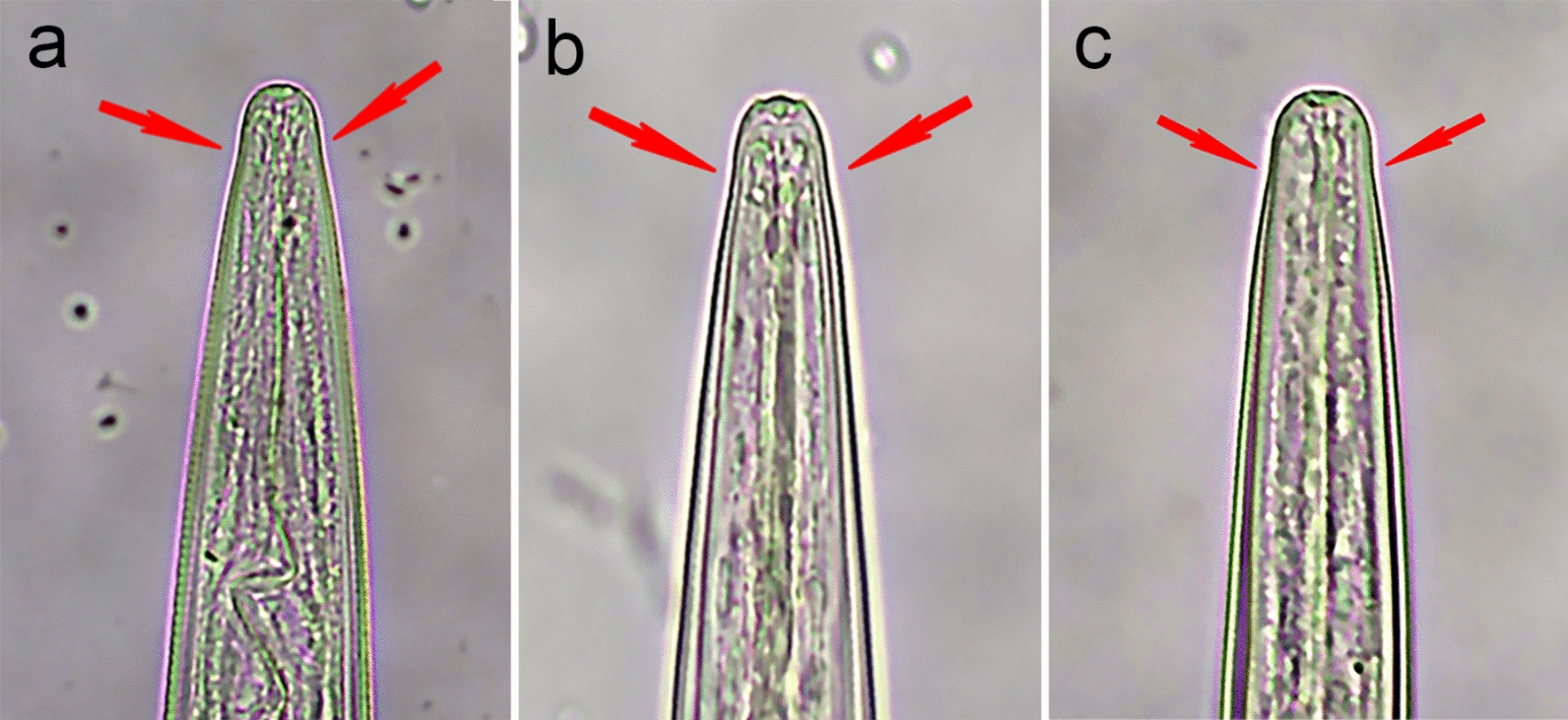
Illustration of the inflexion at the base of the cranial extremity (“shoulder”) commonly seen in infectious *Teladorsagia circumcincta* larvae (**a**, **b**, **c**)

Next, distinction between *Trichostrongylus* spp. and *T. circumcincta* larvae can be made based on the presence of an inflexion (“shoulder”) at the base of the cranial extremity of *T. circumcincta* (Fig. 4), as first described by Lancaster and Hong [37]. In instances where this inflexion can clearly be noted, this morphological characteristic offers an easy distinction between the two types of larvae. However, the authors agree with Roeber and Kahn [7] that this morphological feature is rather subjective and thus easily missed. Besides this, the head of infectious *T. circumcincta* larvae is rather flat and square compared to the rounded shape of *Trichostrongylus* spp. [20], although most likely only noticeable for the experienced parasitologist.

**Fig. 5:**
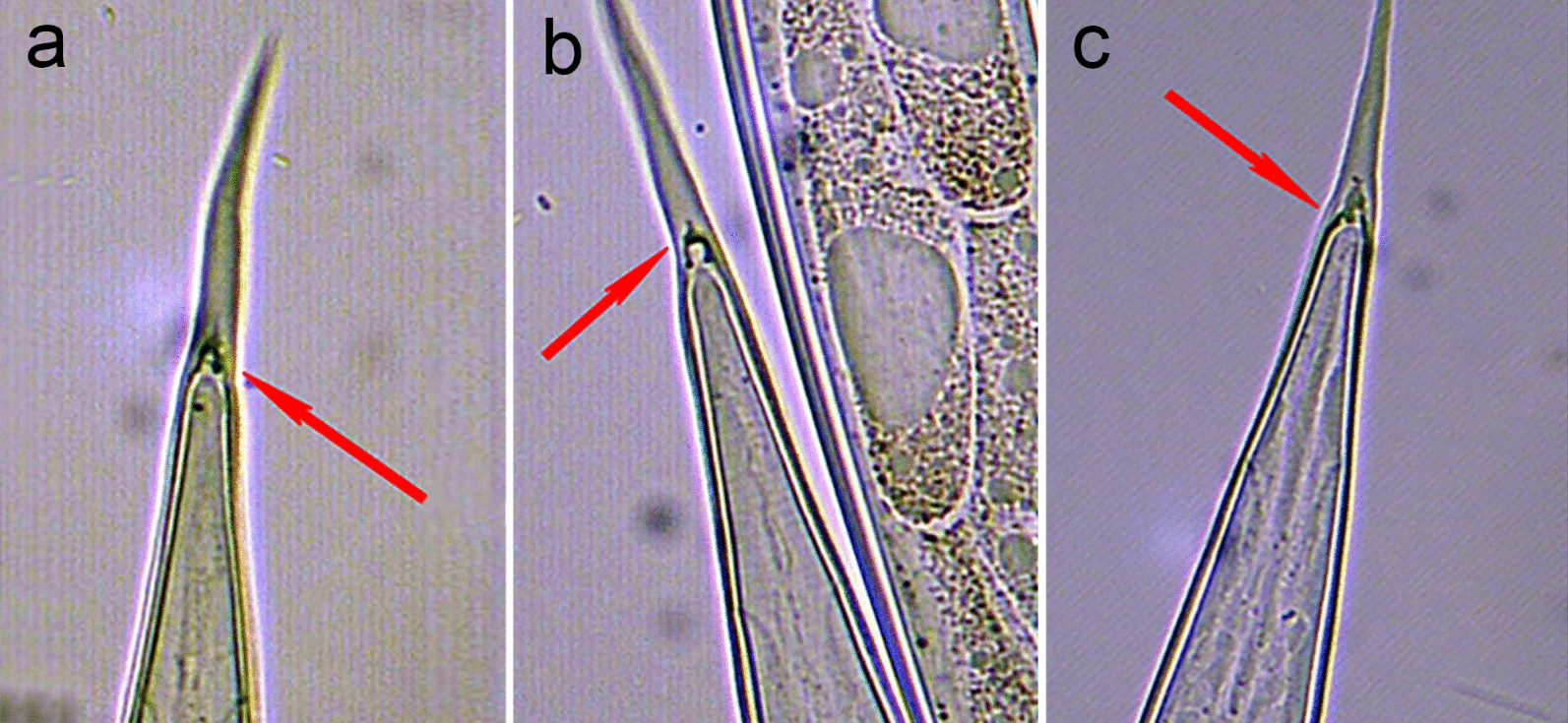
Illustration of the tubercles at the base of the caudal extremity (tail) commonly seen in ensheathed infectious *Trichostrongylus colubriformis* and *Trichostrongylus vitrinus* larvae (**a**, **b**, **c**)

Lastly, most species within the genus *Trichostrongylus* (except *T. axei*) have tubercles (Fig. 5) on their caudal extremity (tail), while these are absent in all other infectious GIN larvae of sheep, including *T. circumcincta* [12, 19, 22, 24]. Usually, these tubercles are readily visible when examining ensheathed larvae under high magnification (×400), and for *T. colubriformis* and *T. vitrinus*, 1–3 tubercles can be noted [22]. Even though this trait cannot be applied for definite differentiation between the two genera within group A, visible tubercles on the caudal extremity allow the larvae to be classified under *Trichostrongylus* spp.

Unfortunately, no definite identification of the respective *Trichostrongylus* species can be achieved through microscopic analysis of their infective larvae. McMurtry [22] originally proposed these to be distinguishable based on detailed assessment of the tubercles seen at high magnification after exsheathment of said larvae, but this was later drawn into question due to significant overlap between the number of tubercles between species [7].

In this study, two larvae morphologically identified as *T. circumcincta* turned out to belong to the genus *Cooperia*. Revision of the morphometric characteristics of said larvae led to the conclusion that a mistake was made when classifying these within their respective preliminary groups. For both specimens, a sheathed tail length exceeding 55 µm was recorded. Within the morphological keys used as a base for this research, only Zajac and Conboy [20] report the upper range of the sheathed tail length of *T. circumcincta* to exceed this value, while other references state the upper limit of the sheathed tail length of the L_3_ of this species as not exceeding 46 µm [12, 19, 27], corresponding to our data. Furthermore, the misidentification which occurred here most likely resulted from the fact that both *T. circumcincta* and *C. curticei* have very similar full body lengths. Statistical analysis showed no significant difference between the average full body length of these two larval types. Besides, considerable overlap was shown between them (Fig. 2c,* T. circumcincta*: 795.32 ± 25.84 µm, *C. curticei*: 813.50 ± 19.35 µm). Lastly, a case can be made that the other morphologically defining characteristics of both these larval types are rather subjective and/or easily missed (e.g., cranial inflexion or the presence of refractile bodies).

Because *Cooperia* spp. was represented only by *C. curticei* larvae within this research, discussion of group B pertains solely to said species and *H. contortus*, and no conclusions were drawn regarding the other ovine *Cooperia* species.

Like the genera in group A, a rudimentary distinction between infectious *C. curticei* (Additional file 2: Figure S3) and *H. contortus* (Additional file 2: Figure S4) larvae in sheep can be made based on full body length measurements. Besides the fact that *Cooperia* spp. L_3_ are commonly reported to be larger than those of *H. contortus* [12, 19, 20, 24], a significant difference in mean body length (Fig. 2c,* H. contortus*: 738.89 ± 14.48 µm, *C. curticei*: 813.50 ± 19.35 µm) was found here. When in doubt, the authors recommend a cut-off of 790 µm for the identification of *C. curticei* [first quartile (Q1) of full body length measurements of *C. curticei* = 791.84 µm].

More notably, sheathed tail morphometry was found to be a reliable trait for the distinction between *H. contortus* and *C. curticei* infectious larvae. A significant difference was found between the sheathed tail lengths of these two types of larvae, and no overlap was demonstrated (Fig. 2b, *H. contortus*: 81.46 ± 2.99 µm, *C. curticei*: 58.75 ± 2.29 µm). This being said, data of the present research might be subject to a sampling bias, as larvae were selected based on specific morphometric characteristics. Larvae (within group B) with a sheathed tail clearly exceeding 65 µm were identified as *H. contortus* and those with a sheathed tail shorter than 65 µm as *Cooperia* spp. For this reason, execution of similar research on blindly sampled larvae might generate different results where considerable overlap can be seen and/or with no significant difference in sheathed tail length, as is commonly reported [12, 19, 20, 24]. Nevertheless, the identities of only two of the larvae microscopically classified within group B turned out to be incorrect. In both cases, *H. contortus* larvae were misidentified as *Cooperia* spp. Revision of the morphometric data of said larvae showed these two to have sheathed tails exceeding 65 µm (the only two within the *Cooperia* spp. dataset), thus providing additional credibility to the above-mentioned morphometric key.

The authors would like to underline that, although sheathed tail length was found to be a good differentiating trait between *C. curticei* and *H. contortus*, this is most likely not the case for all *Cooperia* spp. For example, *Cooperia oncophora* is reported to have a sheathed tail very similar in size to that of *H. contortus* [13, 19, 24], and thus definite differentiation in mixed *Cooperia* samples or samples where *C. oncophora* and *H. contortus* can be found side by side might not be as straightforward if only morphology is used.

One particular differentiating characteristic between *H. contortus* and *Cooperia* spp. (and all other GIN L_3_ for that matter) is commonly reported in the current scientific literature. To this extent, larvae belonging to *Cooperia* spp. are reported to have a pair of oval refractile bodies in their cranial extremities (head) [12, 19, 24]. However, refractile bodies were not clearly observed in any of the *Cooperia* spp. samples within this research. This is in accord with the findings of Dikmans and Andrews [24], who examined pure strains of *C. oncophora* and *C. curticei* in sheep and reported refractile bodies to be clearly visible in infectious *C. oncophora* larvae but much less so in *C. curticei* [24]. Consequently, it can be concluded that this trait is not suitable for the definite differentiation of the larvae within group B.

Finally, as mentioned for the L_3_ classified to have short sheathed tails, the shape of the head of infectious larvae within group B is different as well. *Haemonchus contortus* larvae are reported to have a typical “bullet-shaped” head, while *Cooperia* spp. larvae have a more square/rounded head [12, 19, 20]. Yet again, this is most likely only noticeable for a trained parasitologist.

The identification of the species/genera within the third sheathed tail group (group C) is generally problematic [7, 12, 19]. Microscopic differentiation between *C. ovina* (Additional file 2: Figure S5) and *Oesophagostomum* spp. is most commonly suggested through the counting and the shape of the larvae’s intestinal cells [7], though various sources report different cell counts with significant overlap [12]. An intestinal cell count of 18–22 triangular cells for *Oesophagostomum* spp. and 28–32 rectangular cells for *C. ovina* larvae is believed to be accurate at this time [12]. Nevertheless, intestinal cells can only be clearly perceived in freshly hatched larvae [19], making this characteristic unreliable for samples that have been stored for some time, as was the case here. The number and shape of the intestinal cells of all examined larvae (regardless of the species/genus) were rarely evident within this research.

For the above-mentioned reasons, differentiation between *C. ovina* and *Oesophagostomum* spp. is frequently based on other characteristics, such as a difference in sheathed tail and full body length [12, 19, 20, 24], but, as is the case for most ovine GIN infectious larvae, considerable overlap is reported between these two types of larvae [12, 19, 20, 24]. More importantly, current scientific literature reports the sheathed tail filament length of these larvae to be a reliable trait for identification [12]. *Oesophagostomum* spp. L_3_ are described as having very long filaments constituting up to 70% of the sheathed tail, while for *C. ovina*, filaments typically not exceeding 25% are reported. This being said, on top of being difficult to recognize, no clear description of the transition of sheathed tail to filament exists, making these measurements quite subjective [12, 19].

Genetic analysis unfortunately revealed no *Oesophagostomum* spp. larvae to be present within our samples, and all nine successfully sequenced larvae morphologically identified as potentially representing this genus were wrongly identified, and in fact were *C. ovina*. Misidentification of *Oesophagostomum* spp. larvae here most likely resulted from the unreliable differentiating morphological and morphometric traits explained above. As neither the number and shape of intestinal cells nor sheathed tail filament length could be utilized, identification of *Oesophagostomum* spp. was based mainly on sheathed tail length, and the nine larvae in question thus represent the larvae with the longest sheathed tail within our database. Nevertheless, no larvae with a sheathed tail exceeding 160 µm were observed.

However, as *Oesophagostomum* spp. is rarely encountered in Sardinia [1], the identification of the larvae within this genus was uncertain from the start, and the larvae in question were included solely in an effort to include *Oesophagostomum* spp. within this research. A more sensitive diagnostic technique like deep amplicon sequencing could possibly have detected the presence of *Oesophagostomum* spp. within our samples, as was the case for *Cooperia* spp. in a Canadian study where this particular genus of GIN is uncommon [17].

Finally, the misidentification of *Oesophagostomum* spp. larvae led to the occurrence of additional mistakes within our database. More specifically, underestimation of the upper limit of the sheathed tail length of *C. ovina* resulted in an underestimation of the lower limit of the sheathed tail length of said species as well. Consequently, six larvae having a sheathed tail length between 80 and 100 µm were wrongly identified as *C. ovina* while actually being *H. contortus*. However, current scientific literature does report the upper limit of the sheathed tail length of *H. contortus* to be 78–82 µm [12, 19, 20, 27], clarifying these mistakes.

## Conclusions

It is crucial for the microscopic identification of single infectious GIN larvae to examine each larva in its entirety and thus to take multiple characteristics into account in order to obtain the most accurate diagnosis possible. However, morphometric analysis of the sheathed tail offers a reliable method for preliminary classification of the six most common GIN species/genera in sheep, allowing for their division into three groups of two. Further differentiation can be made based on additional characteristics and measurements. In the case that a larva falling under group A (short sheathed tail) is encountered and no tubercles on the tail or the presence of a cranial inflexion can readily be seen, no definite distinction between *Trichostrongylus* spp. and *T. circumcincta* can be made. At that point, the best course of action would be to make an educated guess based on overall body length. For a larva found to have a medium-sized sheathed tail (group B), a reasonable distinction between *H. contortus* and *C. curticei* can be made based on sheathed tail morphometry. In the case of uncertainty, total body length measurements and the shape of the cranial extremity can aid in reaching a verdict. Besides, *Cooperia* spp. can be identified with certainty if refractile bodies can be noted in the cranial extremity, but this was rarely so in this research. Lastly, distinction between infectious *Oesophagostomum* spp. and *C. ovina* larvae (group C) is generally problematic. Various morphometric and morphological traits have been described for the identification of these two types of larvae (number and shape of intestinal cells, sheathed tail filament length and full body length), but all seem to have significant restrictions.

## Supplementary Information

**Additional file 1: Table S1.** Complete sample list with microscopic identification, molecular identification and sequence numbers reported.

**Additional file 2: Figures S1–S5.** Summary images of the infectious larvae of all five GIN species/genera encountered within this research, including sheathed tail group and morphological and morphometric characteristics using DNA analysis as the gold standard. **Figure S1**. *Trichostrongylus* spp. **Figure S2**. *Teladorsagia circumcincta*. **Figure S3**. *Cooperia curticei*. **Figure S4**. *Haemonchus contortus*. **Figure S5**. *Chabertia ovina*.

## Data Availability

The datasets used and/or analysed during the current study are available from the corresponding author upon reasonable request.

## Abbreviations

ANOVA: Analysis of variance
BLAST: Basic Local Alignment Search Tool
DNA: Deoxyribonucleic acid
dNTP: Deoxynucleotide triphosphate
EPG: Eggs per gram
EU-27: European Union consisting of 27 member states
GIN: Gastrointestinal nematode
ITS-2: Internal transcribed spacer 2
L_3_: Infectious third-stage larvae
PBS: Phosphate-buffered saline
PCR: Polymerase chain reaction
rRNA: Ribosomal RNA
Taq.: *Thermus aquaticus* DNA polymerase
