# Supplementary material for: Practical guide for microscopic identification of infectious gastrointestinal nematode larvae in sheep from Sardinia, Italy, backed by molecular analysis
Source: Parasit Vectors. 2021 Sep 28;14:505. doi: 10.1186/s13071-021-05013-9 (PMC8477562; doi:10.1186/s13071-021-05013-9)
Supplement: Supplementary file 1 — Additional file 1: Table S1. Complete sample list with microscopic identification, molecular identification and sequence numbers reported. [file 13071_2021_5013_MOESM1_ESM.docx]

**Introduction**

This additional file contains Table S1, representing a complete list of the successfully sequenced individual ovine infectious gastrointestinal nematode (GIN) larvae within this research. The table shows the sample number (1-98), the microscopical identification: the identity (species/genus) of each respective larva assigned based on microscopical analysis, the molecular identification: the identity (species) of each larva based on the sequencing of their ITS-2 and partial28S rDNA region.

**Table S1** Complete sample list with microscopical identification, molecular identification and sequence number reported

| Sample number | Microscopical identification | Molecular identification* |
| --- | --- | --- |
| 1 | *Trichostrongylus* spp. | *Trichostrongylus vitrinus* |
| 2 | *Trichostrongylus* spp. | *Trichostrongylus colubriformis* |
| 3 | *Trichostrongylus* spp. | *T. vitrinus* |
| 4 | *Trichostrongylus* spp. | *T. colubriformis* |
| 5 | *Trichostrongylus* spp. | *T. colubriformis* |
| 6 | *Trichostrongylus* spp. | *T. colubriformis* |
| 7 | *Trichostrongylus* spp. | *T. colubriformis* |
| 8 | *Trichostrongylus* spp. | *T. vitrinus* |
| 9 | *Trichostrongylus* spp. | *T. colubriformis* |
| 10 | *Trichostrongylus* spp. | *T. colubriformis* |
| 11 | *Trichostrongylus* spp. | *T. colubriformis* |
| 12 | *Trichostrongylus* spp. | *T. colubriformis* |
| 13 | *Trichostrongylus* spp. | *Trichostrongylus axei* |
| 14 | *Trichostrongylus* spp. | *T. axei* |
| 15 | *Trichostrongylus* spp. | *T. colubriformis* |
| 16 | *Trichostrongylus* spp. | *T. colubriformis* |
| 17 | *Trichostrongylus* spp. | *T. colubriformis* |
| 18 | *Teladorsagia circumcincta* | *T. colubriformis* |
| 19 | *T. circumcincta* | *T. colubriformis* |
| 20 | *T. circumcincta* | *T. vitrinus* |
| 21 | *T. circumcincta* | *T. vitrinus* |
| 22 | *T. circumcincta* | *T. axei* |
| 23 | *T. circumcincta* | *T. axei* |
| 24 | *T. circumcincta* | *T. axei* |
| 25 | *T. circumcincta* | *T. circumcincta* |
| 26 | *T. circumcincta* | *T. circumcincta* |
| 27 | *T. circumcincta* | *T. circumcincta* |
| 28 | *T. circumcincta* | *T. circumcincta* |
| 29 | *T. circumcincta* | *T. circumcincta* |
| 30 | *T. circumcincta* | *T. circumcincta* |
| 31 | *T. circumcincta* | *T. circumcincta* |
| 32 | *T. circumcincta* | *T. circumcincta* |
| 33 | *T. circumcincta* | *T. circumcincta* |
| 34 | *T. circumcincta* | *T. circumcincta* |
| 35 | *Cooperia* spp. | *Cooperia curticei* |
| 36 | *Cooperia* spp. | *C. curticei* |
| 37 | *Cooperia* spp. | *C. curticei* |
| 38 | *Cooperia* spp. | *C. curticei* |
| 39 | *Cooperia* spp. | *C. curticei* |
| 40 | *Cooperia* spp. | *C. curticei* |
| 41 | *Cooperia* spp. | *C. curticei* |
| 42 | *Cooperia* spp. | *C. curticei* |
| 43 | *Cooperia* spp. | *C. curticei* |
| 44 | *Cooperia* spp. | *C. curticei* |
| 45 | *Cooperia* spp. | *C. curticei* |
| 46 | *Cooperia* spp. | *C. curticei* |
| 47 | *Cooperia* spp. | *C. curticei* |
| 48 | *Cooperia* spp. | *C. curticei* |
| 49 | *Cooperia* spp. | *C. curticei* |
| 50 | *Cooperia* spp. | *C. curticei* |
| 51 | *Cooperia* spp. | *C. curticei* |
| 52 | *Cooperia* spp. | *C. curticei* |
| 53 | *Cooperia* spp. | *C. curticei* |
| 54 | *Cooperia* spp. | *C. curticei* |
| 55 | *T. circumcincta* | *C. curticei* |
| 56 | *T. circumcincta* | *C. curticei* |
| 57 | *Haemonchus contortus* | *H. contortus* |
| 58 | *H. contortus* | *H. contortus* |
| 59 | *H. contortus* | *H. contortus* |
| 60 | *H. contortus* | *H. contortus* |
| 61 | *H. contortus* | *H. contortus* |
| 62 | *H. contortus* | *H. contortus* |
| 63 | *H. contortus* | *H. contortus* |
| 64 | *H. contortus* | *H. contortus* |
| 65 | *H. contortus* | *H. contortus* |
| 66 | *H. contortus* | *H. contortus* |
| 67 | *H. contortus* | *H. contortus* |
| 68 | *H. contortus* | *H. contortus* |
| 69 | *H. contortus* | *H. contortus* |
| 70 | *H. contortus* | *H. contortus* |
| 71 | *H. contortus* | *H. contortus* |
| 72 | *H. contortus* | *H. contortus* |
| 73 | *Cooperia* spp. | *H. contortus* |
| 74 | *Cooperia* spp. | *H. contortus* |
| 75 | *Chabertia ovina* | *H. contortus* |
| 76 | *C. ovina* | *H. contortus* |
| 77 | *C. ovina* | *H. contortus* |
| 78 | *C. ovina* | *H. contortus* |
| 79 | *C. ovina* | *H. contortus* |
| 80 | *C. ovina* | *H. contortus* |
| 81 | *C. ovina* | *C. ovina* |
| 82 | *C. ovina* | *C. ovina* |
| 83 | *C. ovina* | *C. ovina* |
| 84 | *C. ovina* | *C. ovina* |
| 85 | *C. ovina* | *C. ovina* |
| 86 | *C. ovina* | *C. ovina* |
| 87 | *C. ovina* | *C. ovina* |
| 88 | *C. ovina* | *C. ovina* |
| 89 | *C. ovina* | *C. ovina* |
| 90 | *Oesophagostomum* spp. | *C. ovina* |
| 91 | *Oesophagostomum* spp. | *C. ovina* |
| 92 | *Oesophagostomum* spp. | *C. ovina* |
| 93 | *Oesophagostomum* spp. | *C. ovina* |
| 94 | *Oesophagostomum* spp. | *C. ovina* |
| 95 | *Oesophagostomum* spp. | *C. ovina* |
| 96 | *Oesophagostomum* spp. | *C. ovina* |
| 97 | *Oesophagostomum* spp. | *C. ovina* |
| 98 | *Oesophagostomum* spp. | *C. ovina* |

*The sequences for each species were deposited in NCBI database with the following accession numbers: MZ323365 (*T. vitrinus*), MZ323366 (*T. colubriformis*), MZ323367 (*T. axei*), MZ323368 (*T. circumcincta*), MZ323369 (*C. curticei*), MZ323370 (*H. contortus*) and MZ323371 (*C. ovina*)
