# Supplementary material for: Practical guide for microscopic identification of infectious gastrointestinal nematode larvae in sheep from Sardinia, Italy, backed by molecular analysis
Source: Parasit Vectors. 2021 Sep 28;14:505. doi: 10.1186/s13071-021-05013-9 (PMC8477562; doi:10.1186/s13071-021-05013-9)

**Introduction**

This additional file contains **Figure S1-S5**, representing summarizing images of the infectious larvae (L_3_) of all five gastrointestinal nematode (GIN) species/genera encountered within this research, including thee images for each larvae with scale (full body, cranial extremity, caudal extremity), sheathed tail group (A, B, C) and brief morphological and morphometrical characteristics obtained using DNA analysis as the gold standard; Fig. S1, *Trichostrongylus* spp.; Fig. S2, *Teladorsagia circumcincta*; Fig. S3, *Cooperia curticei*; Fig. S4, *Haemonchus contortus*; Fig. S5, *Chabertia ovina*.

**Fig. S1** Illustrative figures and summarizing morphological and morphometrical characteristics for infectious *Trichostrongylus* spp. larvae found in the faeces of sheep

**Fig. S2** Illustrative figures and summarizing morphological and morphometrical characteristics for infectious *Teladorsagia circumcincta* larvae found in the faeces of sheep

**Fig. S3** Illustrative figures and summarizing morphological and morphometrical characteristics for infectious *Cooperia* *curticei* larvae found in the faeces of sheep.

*The presence of refractile bodies was unclear in all *C. curticei* larvae identified within this research, hence this might not be an accurate depiction of this trait

**The presence of a sheathed tail filament for larvae classified within Group B was unclear within our hands

**Fig. S4** Illustrative figures and summarizing morphological and morphometrical characteristics for infectious *Haemonchus contortus* larvae found in the faeces of sheep

*The presence of a sheathed tail filament for larvae classified within Group B was unclear within our hands

**Fig. S5** Illustrative figures and summarizing morphological and morphometrical characteristics for infectious *Chabertia ovina* larvae found in the faeces of sheep


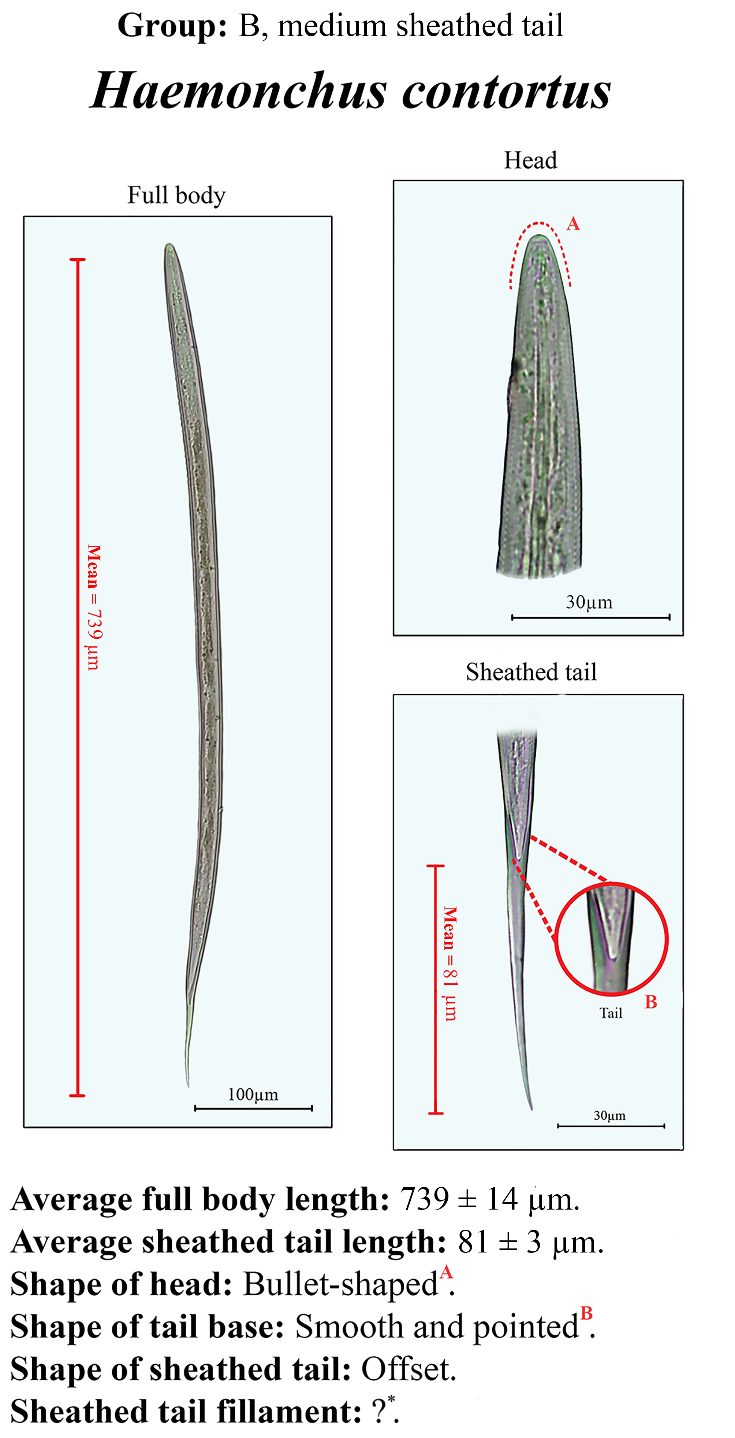

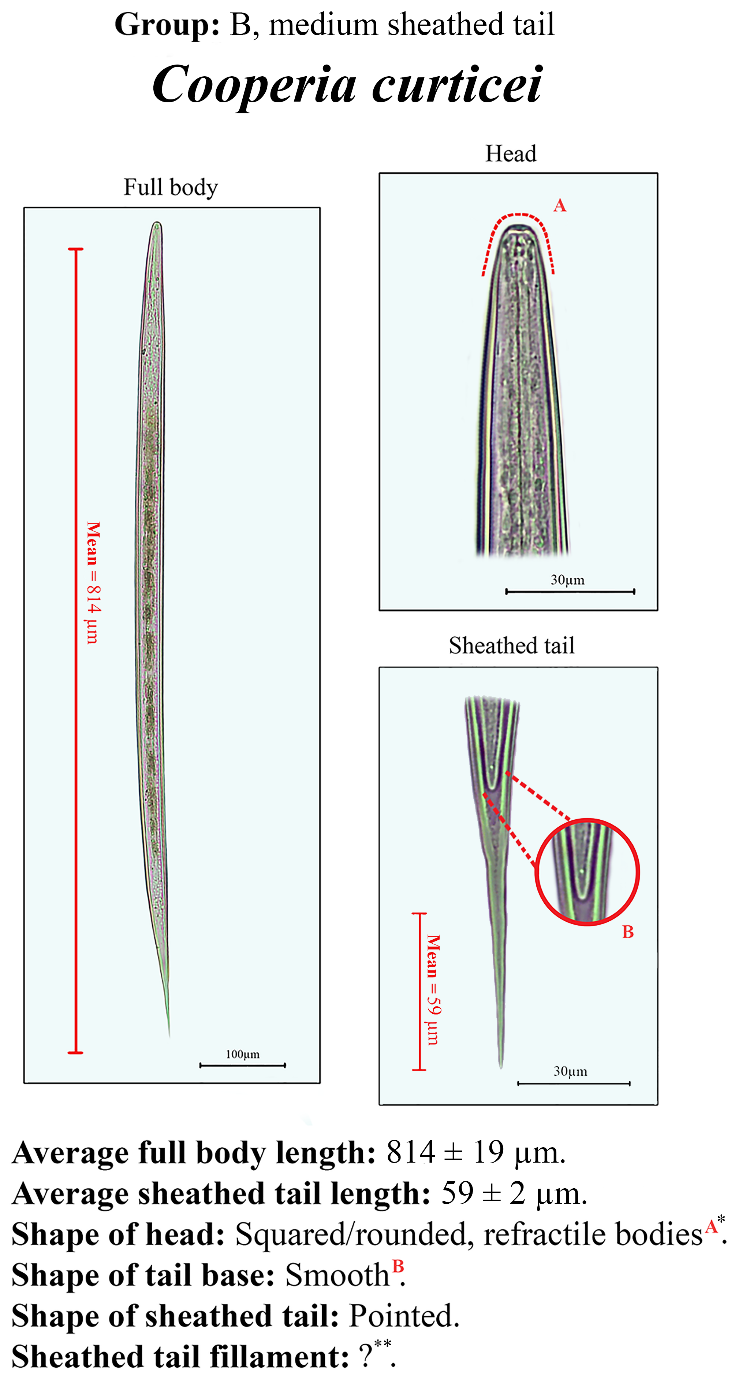

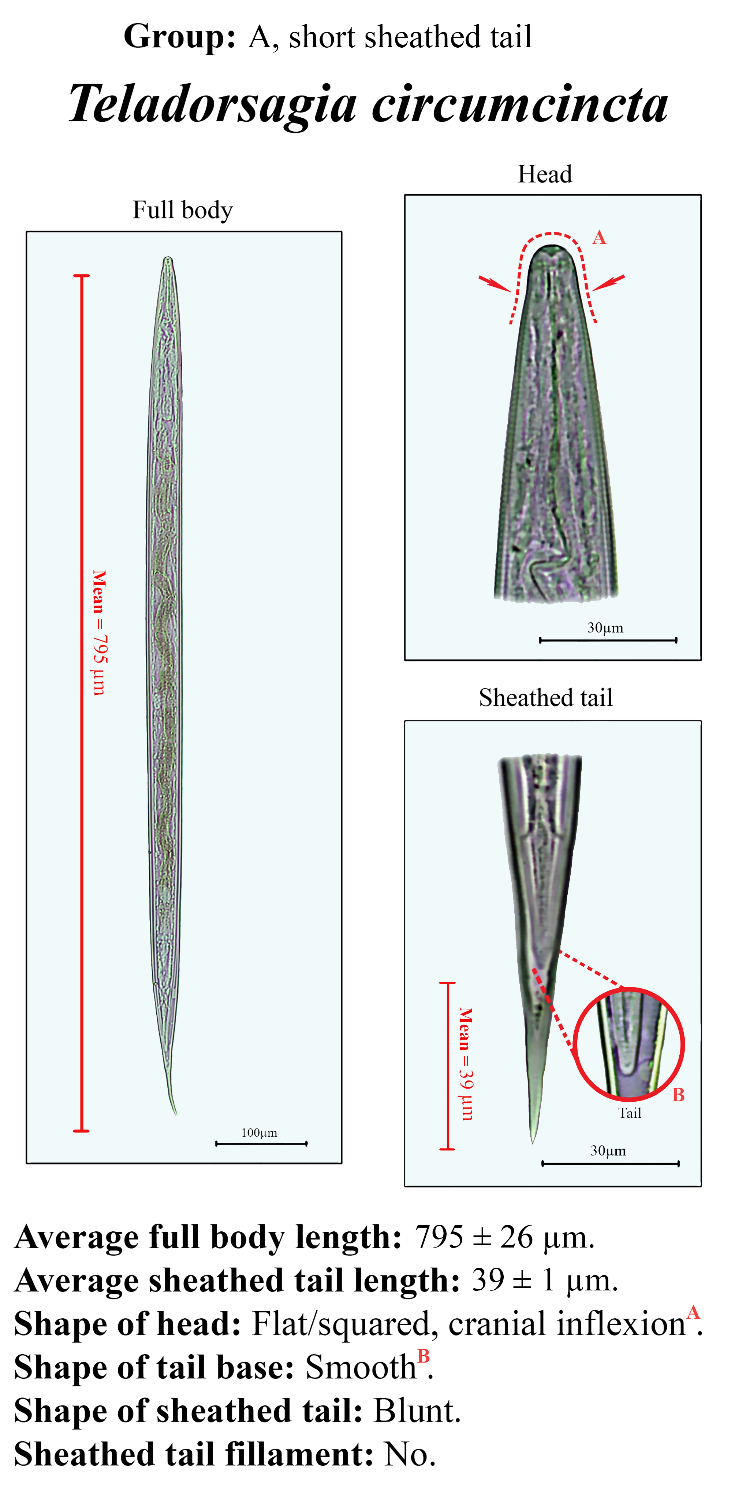

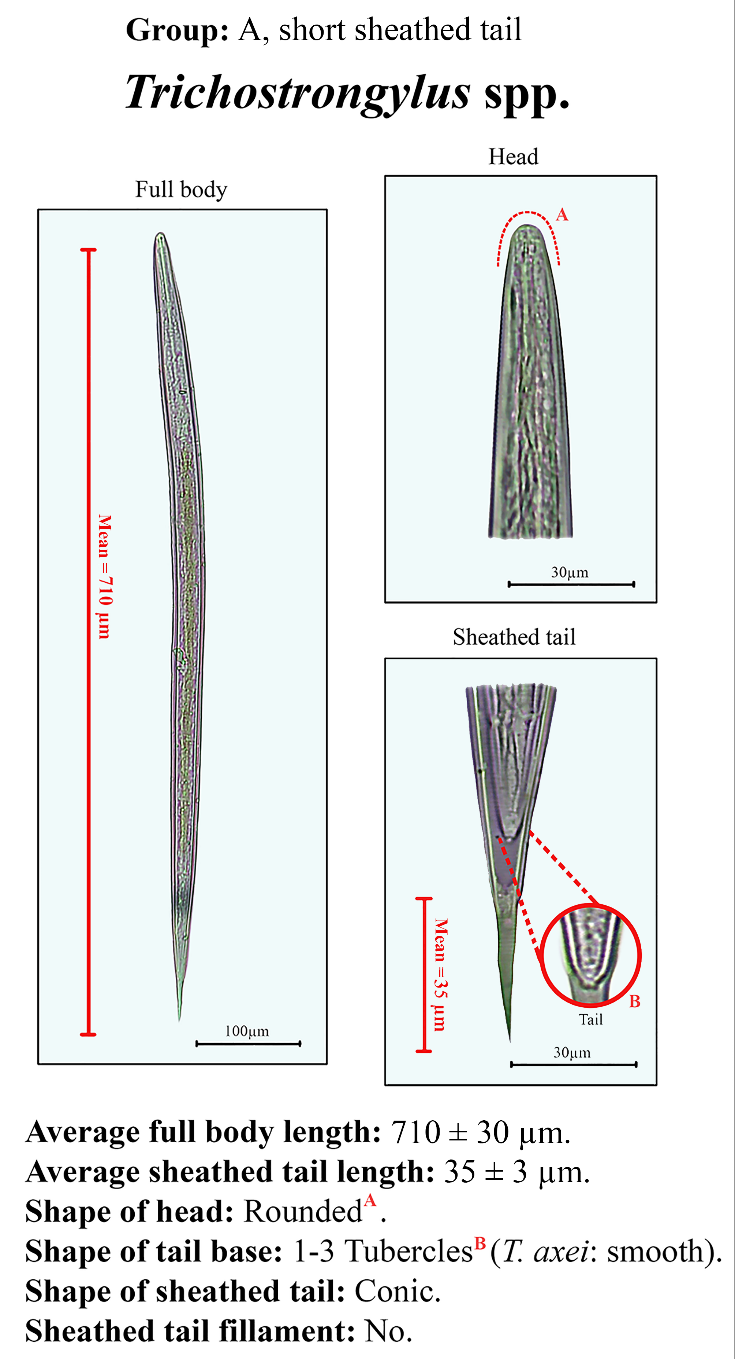

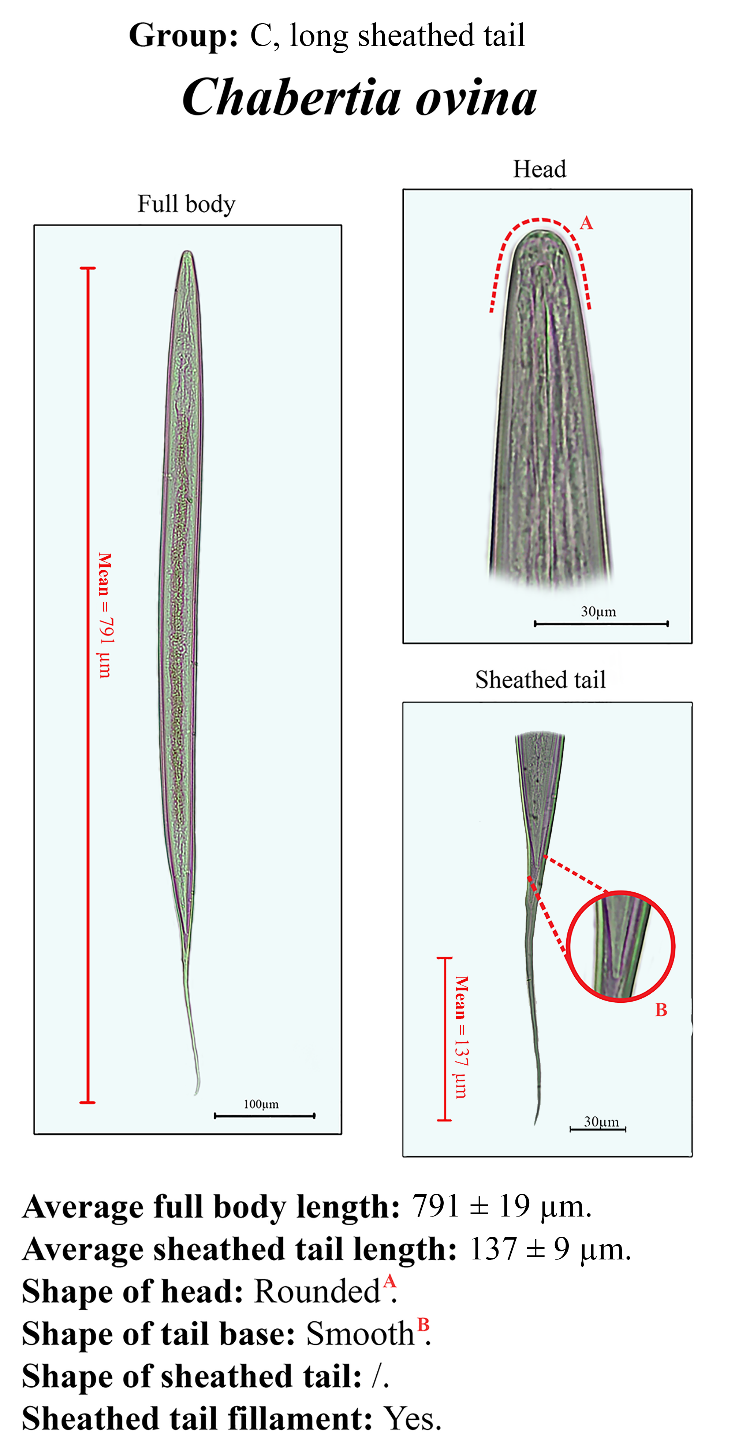

Supplement: Supplementary file 2 — Additional file 2: Figures S1–S5. Summary images of the infectious larvae of all five GIN species/genera encountered within this research, including sheathed tail group and morphological and morphometric characteristics using DNA analysis as the gold standard. Figure S1. Trichostrongylus spp. Figure S2. Teladorsagia circumcincta. Figure S3. Cooperia curticei. Figure S4. Haemonchus contortus. Figure S5. Chabertia ovina. [file 13071_2021_5013_MOESM2_ESM.docx]
